# Chronic Psychologic Stress in Mice Induces Kidney Inflammation

**DOI:** 10.34067/KID.0000001111

**Published:** 2026-01-15

**Authors:** Tara F. Rastegar, Shishir K. Patel, Radhika Kapoor, Ryo Matsuura, Jackson Freiman, Brianna C. Bullock, Paul Welling, Jennifer Pluznick, Chirag R. Parikh, Deidra C. Crews, Sanjeev Noel, Kellie L. Tamashiro, Hamid Rabb

**Affiliations:** 1 Department of Medicine, Johns Hopkins University, Baltimore, Maryland; 2 Department of Psychiatry and Behavioral Sciences, Johns Hopkins University, Baltimore, Maryland; 3 Department of Physiology, Johns Hopkins University, School of Medicine, Baltimore, Maryland

**Keywords:** depression, health status, immunology, kidney disease, quality of life, risk factors, lifestyle medicine, basic science

## Abstract

**Key Points:**

- Chronic stress elevates neutrophil gelatinase-associated lipocalin and cystatin C.
- Chronic stress increases T helper 17 and regulatory T-cell populations in the kidney.
- Chronic stress could increase susceptibility to kidney diseases.

**Background:**

Chronic stress (CS) due to prolonged exposure to negative life events increases the risk of psychiatric illnesses and significantly affects physiologic processes. CS has also been linked to sustained systemic inflammation, resulting in dysregulated immune responses and organ function. We hypothesized that CS would lead to kidney inflammation and tested this in mice.

**Methods:**

Male C57BL/6J mice were subjected to CS by pair-housing them with CD-1 retired breeder mice. Kidney immune cells were isolated and evaluated by spectral flow cytometry. Cytokines were measured using a multiplex assay in kidney tissue and serum. Serum creatinine, cystatin C, and neutrophil gelatinase-associated lipocalin levels were measured.

**Results:**

Mice subjected to CS exhibited greater weight gain than the control group and had reduced fur quality. CS led to a decrease in the percentage of kidney CD4^+^ T cells (54.26%±0.89% versus 59.88%±1.25%, *P* < 0.001) and an increase in T helper 17 (2.3%±0.3% versus 0.83%±0.15%, *P* < 0.001) and regulatory T cells (3.42%±0.45% versus 1.53%±0.21%, *P* < 0.001). There was an increase in the kidney macrophage percentage in CS mice (88.33%±1.16% versus 84.26%±0.73%, *P* < 0.01). TNF-*α* levels were higher in the kidney of stressed mice (8.53±1.18 versus 3.15±1.29 pg/ml, *P* < 0.01). CS led to elevated levels of serum cystatin C (515.9±16.88 versus 456.6±14.79 ng/ml, *P* < 0.05) serum neutrophil gelatinase-associated lipocalin (833.1±282.4 versus 90.58±5.57 ng/ml, *P* < 0.0001).

**Conclusions:**

CS in mice led to kidney inflammation and immunologic changes. These could predispose to acute and CKDs in which inflammation plays a pathogenic role.

## Visual Abstract

**Figure:**
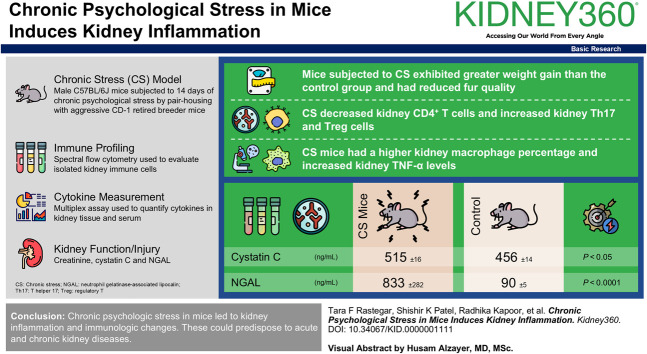

## Introduction

Chronic stress (CS) from prolonged exposure to negative life events increases the risk of psychiatric illnesses, such as mood and anxiety disorders.^1^ CS also leads to significant physical, financial, and emotional repercussions, affecting individuals and society.^2^ CS influences various physiologic processes, including immune responses and organ function.^3^ Previous studies have shown that chronic psychosocial stress can alter immune cell function, leading to inflammation and tissue damage.^4,5^

Inflammatory and functional changes occur in the brain as well as reduced locomotor activity following experimental severe AKI.^6^ Furthermore, meta-analysis of data from AKI patients showed a significant association between AKI and the risk of dementia.^7^ These observations highlight the importance of cross-talk between the kidney and brain. Although the effect of kidney injury on the brain has been studied, it is unclear how CS affects the immune cell population and inflammatory responses in the kidney. This is an important question given that stress-related disorders are increasingly recognized, and inflammation is a key factor in the development and progression of many different kidney diseases.^8^ A previous study demonstrated that acute restraint stress protected from ischemia reperfusion induced AKI^9^; however, the effect of CS on the kidney has not been studied.

Various stress paradigms, including restraint stress and overcrowding stress, have been used to study kidney inflammation; however, these models primarily mimic physical or environmental stressors and do not capture the sustained psychosocial and hierarchy-related stressors that are relevant to human CS.^10,11^ By contrast, chronic social defeat stress (CSDS) is a psychosocial stressor that induces depression, anxiety, anhedonia, and neuroendocrine alterations in mice that can mimic the same pattern in humans.^12,13^ Despite its extensive use in neuroscience research, CSDS has been relatively underexplored in the field of kidney inflammation and dysfunction. A recent study using this model reported stress-induced transcriptomic inflammatory signatures in the kidney and partial reversal with fluoxetine treatment, but they did not assess immune cell composition, cytokine responses, or kidney injury markers.^14^ This gap highlights the need to examine how chronic psychosocial stress affects kidney immune homeostasis and injury pathways.

We hypothesized that CS leads to kidney inflammation and injury. We tested this hypothesis in a mouse model of CSDS. We found that CS results in kidney inflammation, injury, and immunologic changes.

## Methods

### Experimental Animals

Eight week-old male C57BL/6J mice (Jackson Laboratories) were assigned to either a CS group (*n*=10) or control group (*n*=10). Sixteen to 32-week-old male CD-1 retired breeder mice were used as aggressors (Charles River). The mice were allowed to habituate to their new environment for at least 5 days before the start of the CS experiment. To ensure the selection of aggressive resident mice, a screening process was initially conducted. Resident CD-1 mice were allowed to interact with an unfamiliar C57BL/6J mouse for 10 minutes a day for 5 days. The top ten most aggressive CD-1 mice, identified based on their behavior, were selected for this study. All animals were maintained on a 12 hours:12 hours light:dark cycle with *ad libitum* access to water and standard rodent chow (Teklad 2018 Rodent Diet, Inotiv) in a temperature-controlled and humidity-controlled animal housing facility at Johns Hopkins University. All protocols were approved by the Animal Care and Use Committee of the Johns Hopkins University School of Medicine.

### CS Model

**Figure 1:**
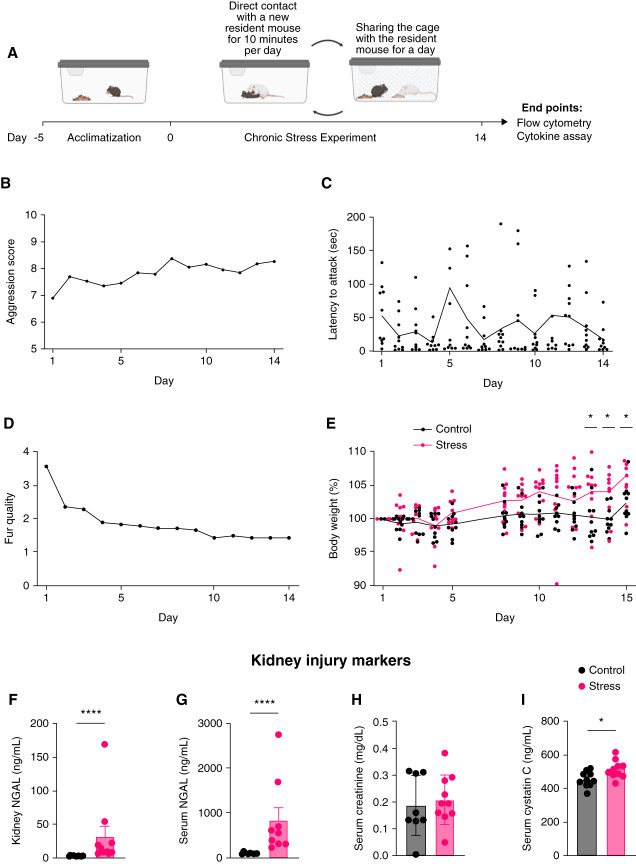
**Experimental design, stress assessment, and kidney injury marker evaluation.** (A) Schematic overview of the experiment design and end points of the study. (B) Body weight change (percentage) of the mice during the CS experiment. (C) Mean aggression score of CD-1 resident mice. (D) Mean fur quality score in stressed and control groups during the CS experiment. (E) Mean latency to attack by CD-1 resident mice. Level of (F) kidney NGAL, (G) serum NGAL, (H) serum creatinine, and (I) serum cystatin C. Data displayed as mean and analyzed by two-way ANOVA with Sidak multiple comparison test (E) and Mann–Whitney *U* test (F–I). *P < 0.05, ****P < 0.0001. CS, chronic stress; NGAL, neutrophil gelatinase-associated lipocalin.

C57BL/6J mice in the CS group were pair-housed with CD-1 resident mice and subjected to CS according to established protocols.^12,13,15^ In brief, each day, a C57BL/6J mouse was introduced to a new CD-1 aggressor for a 10-minute interaction, but the interaction was terminated earlier if aggressive behavior resulted in physical harm to the intruder mouse. After the interaction, the C57BL/6J mouse was placed on the opposite side of a perforated transparent barrier within the home cage of the CD-1 aggressor for 24 hours, allowing for continuous sensory exposure, but no physical contact. This process was repeated with a different CD-1 aggressor each day for 14 consecutive days. Control C57BL/6J mice were cohoused under similar conditions with another control mouse, but without exposure to the CD-1 aggressors. Stressed mice were weighed, and their fur was inspected and scored before each stress period. Fur quality was measured based on a scale of one to four according to coat condition. A score of four indicated a shiny, smooth coat, and a score of one indicated coats that appeared dull and uneven over the whole body. This protocol is based on previous studies.^12^ Aggression of the resident mice was scored on a scale of one to ten based on observed behaviors during the CS period. A score of one indicated no aggression, with no dominant behavior from the resident and no submissive behavior from the intruder. A score of two to four was assigned when the resident exhibited aggressive posturing and consistent dominant behaviors without fighting. A score of five to six indicated that fighting occurred at least once, and a score of seven to ten reflected multiple bouts of fighting throughout the session. On day 15, after the completion of the experiment, all mice were euthanized, and blood samples were drawn retro-orbitally into microcentrifuge tubes. Blood was centrifuged to collect serum, which was then stored at −80°C for further analysis. Kidneys were collected, snap frozen, and stored at −80°C for further analysis (Figure 1A).

### Assessment of Kidney Injury

Serum creatinine was measured to assess kidney function by the Cobas Mira Plus automated analyzer system (Roche) using creatinine measurement reagents (Pointe Scientific Inc.). Neutrophil gelatinase-associated lipocalin (NGAL) and cystatin C were measured using a Multiplex Assay (Millipore Sigma).

### Kidney Immunophenotyping and Intracellular Cytokine Analysis

Kidney mononuclear cells (KMNCs) were isolated as per an established protocol to assess the effects of stress on kidney immune cell infiltration, T-cell activation, and cytokine production.^16^ Approximately 1×10^6^ KMNCs were stained with an antibody cocktail consisting of following antibodies: anti-mouse CD45-Spark Blue 550 (clone: 30.F11), T-cell receptor beta chain-Alexa Fluor 488 (clone: H57-597), CD8*α*-Alexa Fluor 700 (clone: 53-6.7), CD19-APC-Fire 750 (clone: 6D5), F4-80-APC (clone: BM8), NK1.1-BV650 (clone: PK136), CD62L-BV785 (clone: MEL-14), CD44-BV570 (clone: IM7), PD1-BUV805 (clone: 29F.1A12, BD Horizon), T cell immunoreceptor with Ig and ITIM domains (TIGIT)-BV421 (clone:1G9), and CD4-BV480 (clone: RM4-5, BD Horizon).

To measure intracellular cytokines, KMNCs were stimulated with a leukocyte activation cocktail (BD Pharmigen) containing PMA, ionomycin, and brefeldin A before staining with CD45-Spark Blue 550 (clone: 30-F11), T-cell receptor beta chain-Alexa Fluor 488 (clone: H57-597), CD4-BV480 (clone: RM4-5, BD Horizon), CD8-BUV805 (clone: 53-6.7, Invitrogen), IFN*γ*-BV650 (clone: XMG1.2), TNF*α*-BV711 (clone: MP6-XT22), IL17A-BV605 (clone: TC11-18H10.1), IL10-PE/Dazzle594 (clone: JES5-16E3), IL6-PE (clone: MP5-20F3, Invitrogen), and FOXP3-Alexa Fluor 647 (clone: MF-14) using Foxp3/Transcription Factor Staining Buffer Set (eBioscience). Dead cells were labeled with Live/Dead viability dye blue (Invitrogen) and excluded from the analyses. Labeled samples were analyzed with a spectral cytometer, Aurora (Cytek). Live unmixing was performed onboard during data acquisition using Spectroflo (Cytek) and unmixed. FCS files were analyzed with FlowJo 10.10.0 software (BD Biosciences). All antibodies were purchased from BioLegend unless specified otherwise. Gating strategy is shown in Supplemental Figure 1, A and B.

### Multiplex Cytokine Assay

To examine cytokines in serum as well as lysates of kidneys, protein levels of IL-6, IL-10, IL-17A, IFN-*γ*, and TNF-*α* were analyzed using Milliplex Mouse Cytokine/Chemokine Magnetic Bead Panel, Immunology Multiplex Assay (Millipore Sigma). Lysates were sonicated, centrifuged at 4500×*g* for 5 minutes, and stored at −80°C until analysis. The concentration of cytokines and chemokines was normalized using raw protein concentration (measured by Pierce bicinchoninic acid Protein Assay Kit; ThermoFisher Scientific).

### Statistical Analysis

Data are expressed as mean±SEM. Statistical differences were analyzed using the unpaired nonparametric Mann–Whitney U test between two groups or two-way ANOVA with Sidak multiple comparison test, using Prism 10.2.3 (GraphPad Software). Statistical significance was accepted at *P* < 0.05.

## Results

### Assessment of the CS Model

To confirm stress induction, we quantified aggression, monitored attack latency in CD-1 resident mice, and evaluated fur quality in intruder C57BL/6J mice. CD-1 resident mice maintained high aggression scores (seven to nine) throughout the experimental period, indicating persistent aggressive behavior. The fur quality of the intruder mice decreased during the CS experiment (Figure 1, B–D). This CS model has been previously validated demonstrating that 14 days of CS reliably induces anxiety-like and depression-like behaviors, anhedonia, and physiologic changes, including adrenal hypertrophy, splenic hypertrophy, thymic involution, and elevated corticosterone in C57BL/6J mice.^12^

### CS Increased the Body Weight of C57BL/6J Mice

Both groups gained weight over time; however, C57BL/6J mice subjected to CS exhibited a significantly greater increase compared with controls (Figure 1E), with differences reaching statistical significance from day 13 onward (*P* < 0.05).

### CS Increased Kidney Injury Markers

To assess the effect of CS on kidney injury, we measured creatinine, cystatin C, and NGAL in the serum of the mice. Serum creatinine levels were not significantly different between mice in stressed and control groups (0.21±0.03 versus 0.19±0.04 mg/dl; Figure 1H). However, stressed mice exhibited significantly elevated serum levels of cystatin C (515.96±16.88 versus 456.66±14.79 ng/ml, *P* < 0.05) and NGAL (833.16±282.4 versus 90.58±65.57 ng/ml, *P* < 0.0001; Figure 1, G and I). Consistent with these findings, NGAL levels in kidney tissue lysates were also increased in the stressed group (31.60±16.01 versus 2.39±0.21 ng/ml, *P* < 0.0001; Figure 1F).

### CS Changed Immune Cell Composition in the Kidney

**Figure 2:**
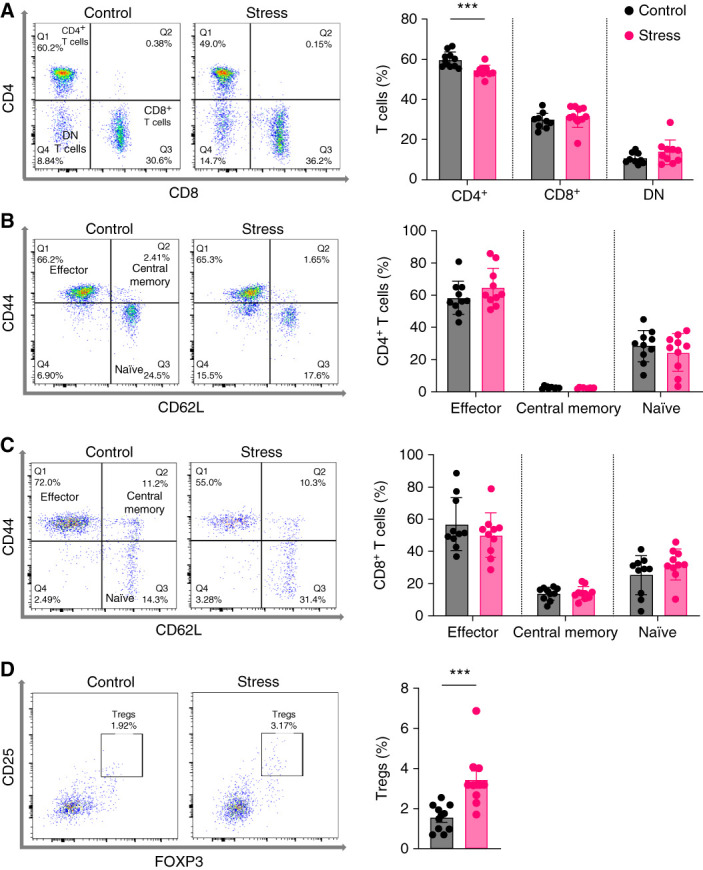
**Flow cytometry assessment of kidney T-cell populations.** (A) Representative flow cytometry images showing the gating strategy for assessment of T-cell subgroups and percentage of CD4^+^, CD8^+^, and DN T cells among total T cells in the control and stressed groups. (B) Representative flow cytometry images showing the gating strategy for assessment of CD4^+^ T-cell subsets and percentage of effector, central memory, and naïve CD4^+^ T cells. (C) Representative flow cytometry images showing the gating strategy for assessment of CD8^+^ T-cell subsets, and percentage of effector, central memory, and naïve CD8^+^ T cells. (D) Representative flow cytometry images showing the gating strategy for assessment of Tregs and percentage of Tregs. Data displayed as mean±SEM and analyzed by Mann–Whitney *U* test. ***P < 0.001. DN, double-negative; Treg, regulatory T cell.

Comprehensive immunophenotypic analysis using multiparameter spectral flow cytometry revealed that the percentage (54.26%±0.90% versus 59.88%±1.25%, *P* < 0.001) and absolute number (58,475±12,982 versus 105,011±13,507, *P* < 0.05) of CD4^+^ T cells in the kidneys of stressed mice were significantly reduced compared with control mice. The percentage and absolute number of CD8^+^ T cells (31.49%±1.73% versus 29.31%±1.21% and 32,110±6363 versus 52,222±7774) and double-negative T cells (13.80%±1.92% versus 10.46%±0.83% and 17,577±6647 versus 19,103±3458) remained unchanged (Figure 2A and Supplemental Figure 2A).

We evaluated the effect of CS on T-cell memory phenotype by assessing CD62L and CD44 expression. We observed a significant reduction in the absolute number of effector CD4^+^ T cells in the CS group (58.64%±3.27% versus 64.90%±3.82%; 35,569±8891 versus 68,517±9510, *P* < 0.05). CS did not affect either the percentage or the absolute numbers of naïve (28.57%±3.11% versus 24.41%±3.71%; 15,227±3266 versus 25,108±4434) or central memory (2.62%±0.22% versus 2.30%±0.17%; 1508±313.90 versus 2486±432.80) CD4^+^ T cells. Similarly, there was no change in effector memory (50.46%±4.37% versus 57.27%±5.23%; 16.643±4030 versus 30,213±5476), central memory (14.30%±1.34% versus 13.99%±1.27%; 4349±842.3 versus 7368±1234), or naïve (32.24±3.01 versus 25.52±3.84; 10,072%±2216% versus 13,035%±2360%) CD8^+^ T cells (Figure 2, B and C and Supplemental Figure 2, C and D). CS increased both the percentage and absolute number of CD4^+^ CD25^+^ FOXP3^+^ regulatory T cell (Treg) in the kidneys of stressed mice compared with control mice (3.42%±0.45% versus 1.53%±0.21%, *P* < 0.001; and 1673±284.4 versus 745.8±158.8, *P* < 0.05; Figure 2D and Supplemental Figure 2B).

**Figure 3:**
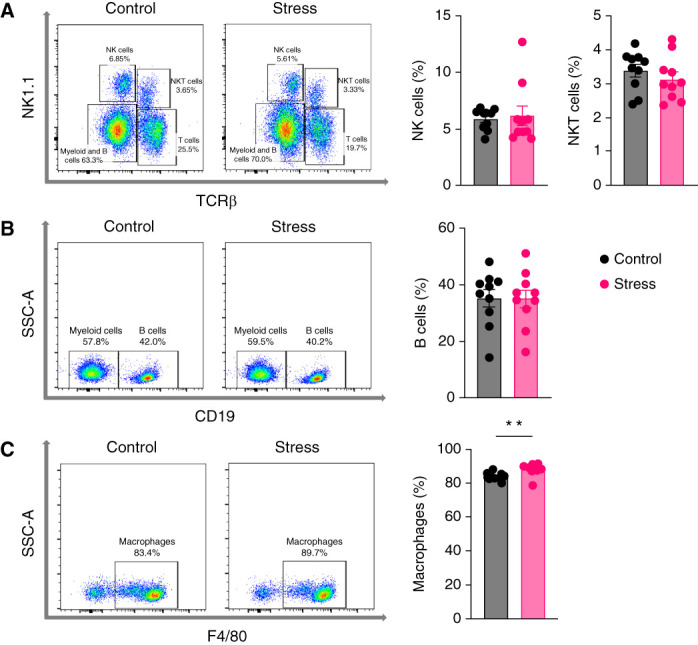
**Flow cytometry assessment of kidney NK, NKT, B cells and macrophages.** (A) Representative flow images show the gating strategy for assessment NK cells and NKT cells and percentage of NK cells and NKT cells among CD45^+^ cells. (B) Representative flow images show the gating strategy for assessment of CD19^+^ B cells and percentage of CD19^+^ B cells among CD45^+^, NK1.1^−^, TCR*β*^−^ cells. (C) Representative flow images show the gating strategy for assessment of F4/80^+^ macrophages, and percentage of F4/80^+^ macrophages among CD45^+^, NK1.1^−^, TCR*β*^−^, CD19^−^ cells. Data displayed as mean±SEM and analyzed by Mann–Whitney *U* test. **P < 0.01. NK, natural killer; NKT, natural killer T; SSC-A, side scatter area; TCR*β*, T-cell receptor beta chain.

Analysis of other lymphoid populations showed a significant reduction in absolute numbers of natural killer (NK) T cells (18,111±4790 versus 29,797±4417, *P* < 0.05), although the percentage of NK T cells (3.14±0.21 versus 3.39±0.18) was not affected. We found no significant difference in the percentage and absolute number of NK cells (6.16%±0.85% versus 5.88%±0.29%; 35,491±9963 versus 51,473±7140) or B cells (35.00%±3.11% versus 35.27%±3.09%; 132,589±30,879 versus 214,508±32,356) following CS (Figure 3, A and B and Supplemental Figure 2, E–G).

Flow cytometric analysis revealed an increased percentage of CD45^+^ F4/80^+^ macrophages (88.33±1.16 versus 84.26±0.73, *P* < 0.01). No differences were observed in the absolute number of macrophages (219,513±50,375 versus 340,841±52,304; Figure 3C and Supplemental Figure 2H).

### Effect of CS on Kidney Immune Checkpoint Molecules

**Figure 4:**
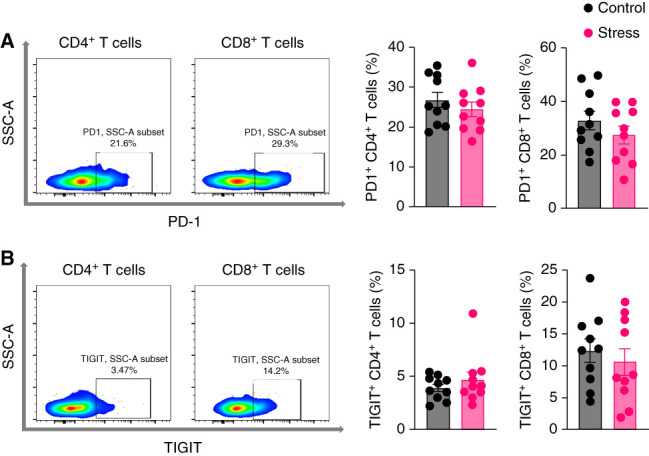
**Flow cytometry assessment of kidney T cell immune checkpoint inhibitors.** (A) Representative flow cytometry images show the gating strategy for assessment of PD-1 expression in CD4^+^ and CD8^+^ T cells and percentage of PD-1 expressing CD4^+^ and CD8^+^ T cells. (B) Representative flow cytometry images show the gating strategy for assessment of TIGIT expression in CD4^+^ and CD8^+^ T cells and percentage of TIGIT expressing CD4^+^ and CD8^+^ T cells. Data displayed as mean±SEM and analyzed by Mann–Whitney *U* test. PD-1, programmed cell death protein-1; TIGIT, T cell immunoreceptor with Ig and ITIM domains.

To assess the effect of CS on the expression of immune checkpoint molecules in kidney T cells, we quantified the expression of programmed cell death protein-1 (PD-1) and TIGIT on CD4^+^ and CD8^+^ T-cell populations. We observed a significant reduction in the absolute number of CD4^+^ T cells expressing PD-1 in the stressed group (14,344±3430 versus 28,700±4664, *P* < 0.05); however, the percentage of PD-1^+^ cells within the CD4^+^ T cells remained unchanged (24.57±1.82 versus 26.84±1.96). There was no difference in the percentage and absolute number of PD1-expressing CD8^+^ T cells (27.55%±3.32% versus 32.98%±3.54%; 9367±2572 versus 17,824±3774). In addition, no difference was observed in TIGIT expression in CD4^+^ (4.70%±0.76% versus 3.93%±0.35%; 2585±613.7 versus 4189±775.7) and CD8^+^ T cells (10.60%±2.07% versus 12.41%±1.84%; 3321±1014 versus 6511±1313; Figure 4, A and B and Supplemental Figure 3, A–D).

### CS Modulated Cytokine Production by T Cells in the Kidney

**Figure 5:**
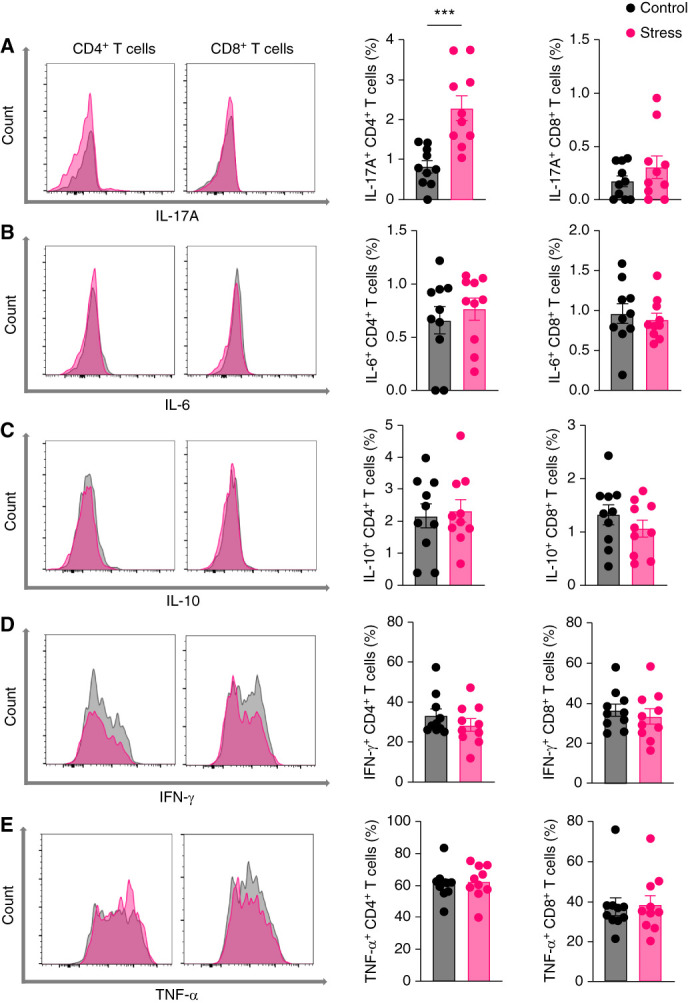
**Cytokine expression in CD4**^**+**^ **and CD8**^**+**^ **T cells.** Representative histograms, percentages of cytokine-expressing CD4^+^ and CD8^+^ T cells. (A) IL-17, (B) IL-6, (C) IL-10, (D) IFN-*γ*, and (E) TNF-*α*. Data displayed as mean±SEM and analyzed by Mann–Whitney *U* test. ***P < 0.001.

We next assessed the expression of stress-related cytokines in CD4^+^ and CD8^+^ T cells. Specifically, we analyzed intracellular levels of IL-6, IL-10, IL-17A, IFN-*γ*, and TNF-*α*. Among CD4^+^ T cells, a significant increase in IL-17A (T helper 17 [Th17]) production was observed in percentage and absolute number in the stressed group (2.30%±0.31% versus 0.83%±0.15%, *P* < 0.001; and 1198±242.5 versus 347.4±56.78, *P* < 0.01). No significant differences were detected in IL-6 (0.76%±0.10% versus 0.66%±0.13%, and 413.8±77.68 versus 314.9±76.81), IL-10 (2.33%±0.35% versus 2.17%±0.38%, and 1128±208.3 versus 1051±218.4), IFN-*γ* (28.53%±3.16% versus 33.21%±3.28% and 14,039±2452 versus 14,479±1207), and TNF-*α* (62.21%±3.34% versus 60.96%±3.13% and 30,557±4314 versus 26,699±1825) among CD4^+^ T cells. No significant difference in IL-17A (0.31%±0.10% versus 0.17%±0.05%, and 168.8±56.22 versus 82.47±29.96), IL-6 (0.88%±0.08% versus 0.96%±0.12% and 475.8±71.46 versus 450.7±74.06), IL-10 (1.07%±0.16% versus 1.33%±0.19% and 584.7±126.1 versus 612.9±120.2), IFN-*γ* (33.43%±3.9% versus 36.69%±0.32% and 18,543±3543 versus 17,005±2805), and TNF-*α* (38.35%±4.66% versus 37.08%±4.57% and 21,074±4073 versus 16,163±2313) among CD8^+^ T cells (Figure 5, A–E and Supplemental Figure 3, E–N).

### CS Increased TNF-*α* in the Kidney

**Figure 6:**
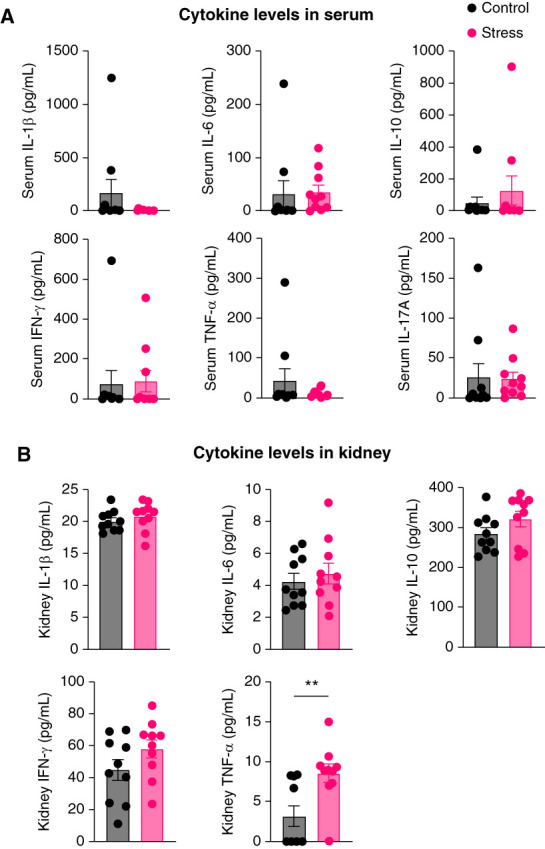
**Level of stress-related cytokines in the kidney lysates and serum.** (A) Cytokine levels in serum. IL-1*β*, IL-6, IL-10, IFN-*γ*, TNF-*α*, and IL-17A. (B) Cytokine levels in kidney. IL-1*β*, IL-6, IL-10, IFN-*γ*, and TNF-*α*. Data are presented as mean±SEM and analyzed by Mann–Whitney *U* test. **P < 0.01.

We further assessed systemic and kidney-specific inflammatory response to CS by measuring IL-1*β*, IL-6, IL-10, IL-17A, TNF-*α*, and IFN-*γ* in the serum and whole kidney tissue lysates. We did not observe any difference in serum IL-1*β* (3.75±2.41 versus 169.4±125.7 pg/ml), IL-6 (36.14±12.53 versus 33.04±24.03 pg/ml), IL-10 (126.2±91.64 versus 48.15±37.71 pg/ml), IL-17A (24.32±8.45 versus 26.43±16.80 pg/ml), TNF-*α* (10.27±2.83 versus 44.67±29.11 pg/ml), and IFN-*γ* (90.28±53.43 versus 74.23±68.75 pg/ml) levels. However, there was a significant increase in TNF-*α* in the kidney of the stressed mice compared with control mice (3.15±1.29 versus 8.53±1.18 pg/ml, *P* < 0.01). The level of IL-1*β* (20.81±0.7 versus 20.10±0.50 pg/ml), IL-6 (4.75±0.66 versus 4.23±0.50 pg/ml), IL-10 (322.8±19.54 versus 285.1±14.60 pg/ml), and IFN-*γ* (58.17±5.69 versus 44.82±6.54 pg/ml) in the kidney was not affected after CS (Figure 6, A and B).

## Discussion

We examined the effect of CS on kidney inflammation and immunology using a well-established CS mouse model. CS led to an increase in proinflammatory pathways, characterized by elevated Th17 cells, higher TNF-*α* level, and increased kidney macrophage infiltration. Furthermore, we observed upregulation of both plasma and kidney NGAL, along with elevated serum cystatin C. However, CS also enhanced the frequency of Tregs while reducing the overall CD4^+^ T-cell population.

The decrease in CD4^+^ T cells and the increase in proportion of Th17 and Tregs observed in this study suggest a complex interplay between different immune cell types under stress conditions. Tregs have been shown to reduce depression-like behavior in mice subjected to chronic restraint stress, indicating that they reduce the adverse effects of stress on the body.^17^ Several studies have shown that Tregs can suppress the development of chronic inflammatory diseases such as lupus and rheumatoid arthritis.^18,19^ Th17 cells are known for their role in recruiting neutrophils and promoting inflammation, while Tregs are crucial for maintaining immune tolerance and suppressing excessive immune responses.^20^ Previous studies have shown that repeated exposure to social defeat stress causes an increase in peripheral myeloid cells, such as neutrophils and monocyte/macrophages.^21^ Macrophages can also be involved in tissue repair and resolution of inflammation.^22^ We observed that CS increased macrophage proportion in the kidney tissue which can be indicative of heightened tissue remodeling and inflammatory activity.

Our observation showing increased TNF-*α* in the kidneys of stressed mice suggests a proinflammatory milieu in the kidney. Previous studies on repeated social defeat stress have shown increased TNF-*α* expression in the liver and prefrontal cortex of the brain, indicating elevated inflammation in chronically stressed mice across multiple organs.^23,24^ Such a proinflammatory environment is speculated to increase the susceptibility of the kidney to secondary insults such as ischemia-reperfusion and nephrotoxicity in stressed individuals.^25,26^

We observed that CS led to significant changes in kidney injury biomarkers, specifically cystatin C and NGAL. These biomarkers are indicative of kidney damage and dysfunction.^27^ The increase in NGAL levels further strengthens the notion that CS may exacerbate kidney injury, potentially through mechanisms involving inflammation and immune cell activation.

This study was conducted in a mouse model of CS, which may not fully represent the complexity of human CS, and only male mice were used. In addition, although the study identified immune alterations, it did not explore mechanisms, such as the role of stress hormones on immune cell alterations. We also did not study kidney disease models, which could demonstrate what the clinically significant effects were of the changes we observed. Finally, our flow cytometry panel did not include markers necessary to distinguish resident versus infiltrating macrophage subsets; therefore, future studies incorporating additional phenotypic markers will be necessary to define the specific macrophage subsets altered by CS.

In conclusion, these results demonstrate that CS led to elevated TNF-*α* levels, T-cell IL-17 levels, and increased NGAL, an important biomarker of kidney injury in serum and kidney. These results have important clinical implications as CS can reduce the threshold for developing AKI and worsen established CKDs. Future studies are required to assess the effects of CS on kidney disease models as well as study biologic markers of CS in clinical kidney disease cohorts.

## Data Availability

Original data generated for the study will be made available upon reasonable request to the corresponding author. Data Type: Raw Data/Source Data, Research Protocols, and Statistical Analysis Plan. Reason for Restricted Access: All data presented in this article are available upon request to the corresponding author.

## Declarative Statements

All animal experiments were conducted in accordance with the NIH Guide for the Care and Use of Laboratory Animals or an equivalent standard that meets or exceeds the ethical and welfare requirements outlined in the NIH Guide. All protocols were approved by the appropriate institutional animal care and use committee.

## Supplemental Material

Supplemental Figure 1. Gating strategy for the surface and intracellular markers for kidney T cells. Isolated KMNCs were analyzed with a spectral flow cytometer as described in Methods. After single cell gating, lymphocytes were identified with side scatter area and CD45^high^ gating. (A) Panel 1 for investigating leukocyte subsets. (B) Panel 2, for investigating the intracellular cytokines in T-cell subsets.

Supplemental Figure 2. Assessment of absolute number of leukocytes. (A) Absolute number of T-cell subsets. (B) Absolute number of Tregs. (C) Absolute number of effector, central memory, and naïve CD4^+^ T cells. (D) Absolute number of effector, central memory, and naïve CD8^+^ T cells. (E) Absolute number of NKT cells. (F) Absolute number of NK cells. (G) Absolute number of B cells. (H) Absolute number of macrophages. Data displayed as mean±SEM and analyzed by Mann–Whitney *U* test. **P* < 0.05.

Supplemental Figure 3. Assessment of the absolute number of immune checkpoint inhibitor and cytokine-expressing T cells. Absolute number of (A) CD4^+^ and (B) CD8^+^ T cells expressing programmed cell death protein-1. Absolute number of (C) CD4^+^ and (D) CD8^+^ T cells expressing TIGIT. Absolute number of cytokine-expressing (E) IL-17, (F) IL-6, (G) IL-10, (H) IFN-*γ*, (I) TNF-*α* CD4^+^ T cells, and (J) IL-17, (K) IL-6, (L) IL-10, (M) IFN-*γ*, (N) TNF-*α* CD8^+^ T cells. Data are presented as mean±SEM and analyzed by Mann–Whitney *U* test. **P* < 0.05, ***P* < 0.01.
